# Intratumoral administration of STING-activating nanovaccine enhances T cell immunotherapy

**DOI:** 10.1136/jitc-2021-003960

**Published:** 2022-05-26

**Authors:** Xiaoyi Jiang, Jian Wang, Xichen Zheng, Zhida Liu, Xinyu Zhang, Yuwei Li, Jonathan Wilhelm, Jun Cao, Gang Huang, Jinlan Zhang, Baran Sumer, Jayanthi Lea, Zhigang Lu, Jinming Gao, Min Luo

**Affiliations:** 1 Institute of Pediatrics, Children's Hospital of Fudan University, and Shanghai Key Laboratory of Medical Epigenetics, International Co-laboratory of Medical Epigenetics and Metabolism, Ministry of Science and Technology, Institutes of Biomedical Sciences, Fudan University, Shanghai, China; 2 Department of Pharmacology, Harold C. Simmons Comprehensive Cancer Center, University of Texas Southwestern Medical Center, Dallas, Texas, USA; 3 Department of Immunology, Tianjin Medical University Cancer Institute and Hospital, National Clinical Research Center for Cancer, Key Laboratory of Cancer Prevention and Therapy, Key Laboratory of Cancer Immunology and Biotherapy, Tianjin’s Clinical Research Center for Cancer, Tianjin, China; 4 Shanxi Academy of Advanced Research and Innovation, Taiyuan, Shanxi, China; 5 The Fifth People’s Hospital of Shanghai, Fudan University, Shanghai, China; 6 Department of Otolaryngology, Harold C. Simmons Comprehensive Cancer Center, University of Texas Southwestern Medical Center, Dallas, Texas, USA; 7 Department of Obstetrics and Gynecology, Harold C. Simmons Comprehensive Cancer Center, University of Texas Southwestern Medical Center, Dallas, Texas, USA; 8 Shanghai Institute of Infectious Diseases and Biosecurity, Shanghai Medical College, Fudan University, Shanghai, China

**Keywords:** vaccination, lymphocytes, tumor-infiltrating, translational medical research, immunotherapy

## Abstract

**Background:**

Cancer vaccines are able to achieve tumor-specific immune editing in early-phase clinical trials. However, the infiltration of cytotoxic T cells into immune-deserted tumors is still a major limiting factor. An optimized vaccine approach to induce antigen-specific T cells that can perform robust tumor infiltration is important to accelerate their clinical translation. We previously developed a STING-activating PC7A nanovaccine that produces a strong anti-tumor T cell response on subcutaneous injection. This study systematically investigated the impact of administration methods on the performance of nanovaccines.

**Methods:**

Tumor growth inhibition by intratumoral delivery and subcutaneous delivery of nanovaccine was investigated in TC-1 human papillomavirus-induced cancer model and B16-OVA melanoma model. Nanovaccine distribution in vivo was detected by clinical camera imaging, systemic T cell activation and tumor infiltration were tested by in vivo cytotoxicity killing assay and flow cytometry. For mechanism analysis, T cell recruitment was investigated by in vivo migration blocking assay, multiplex chemokine array, flow cytometry, RT-qPCR, chemotaxis assay and gene knockout mice.

**Results:**

Nanovaccine administration was found to alter T cell production and infiltration in tumors. Intratumoral delivery of nanovaccines displayed superior antitumor effects in multiple tumor models compared with subcutaneous delivery. Mechanistic investigation revealed that intratumoral administration of the nanovaccine significantly increased the infiltration of antigen-specific T cells in TC-1 tumors, despite the lower systemic levels of T cells compared with subcutaneous injection. The inhibition of tumor growth by nanovaccines is primarily dependent on CD8^+^ cytotoxic T cells. Nanovaccine accumulation in tumors upregulates CXCL9 expression in myeloid cells in a STING dependent manner, leading to increased recruitment of IFNγ-expressing CD8^+^ T cells from the periphery, and IFNγ reciprocally stimulates CXCL9 expression in myeloid cells, resulting in positive feedback between myeloid-CXCL9 and T cell-IFNγ to promote T cell recruitment. However, the STING agonist alone could not sustain this effect in the presence of a systemic deficiency in antigen-specific T cells.

**Conclusions:**

Our results demonstrate that intratumoral administration of PC7A nanovaccine achieved stronger antitumor immunity and efficacy over subcutaneous injection. These data suggest intratumoral administration should be included in the therapeutic design in the clinical use of nanovaccine.

## Background

Checkpoint immunotherapy (eg, pembrolizumab) has revolutionized cancer care with durable responses in patients with immunogenic tumors. However, the majority of patients with cancer fail to benefit from checkpoint therapy due to inadequate cancer-specific T cell production and infiltration into tumors.^1–3^ Therapeutic vaccines that generate antigen-specific T cells have long been sought after to boost antitumor immunity.^4–6^ Recent progress with rapid identification of tumor neoantigens offers a broad repertoire of cancer-specific targets for vaccine development.^5 7^ Early-phase clinical trials in patients with advanced melanoma or glioblastoma show that neoantigen-based vaccines generate antigen-specific T cell responses which is safe and potentially effective.^8–11^ Further development of vaccine technology that converts tumor-specific antigens/neoantigens into efficacious cancer therapy in the clinic is urgently needed.^12^

Current vaccine formulations incorporate adjuvants to activate innate immune pathways to enhance Th1 and cytotoxic T cell responses.^8 13^ Nanotechnology can play a unique role in establishing a multifunctional platform that integrates antigen delivery with innate stimulation.^13 14^ Nanoparticles less than 100 nm in diameter can efficiently drain to peripheral lymph nodes (LNs) after subcutaneous or intradermal delivery.^15 16^ Previously, we reported a STING-activating nanovaccine (<50 nm) by a simple physical mixture of antigen peptides with a synthetic polymeric nanoparticle, PC7A NP(nanoparticle).^17^ PC7A enhances antigen delivery and cross-presentation and stimulates the STING- type I IFN pathway to boost T cell response against tumor. In STING^gt/gt^ and IFN-α/βR^-/-^ mice, majority of the CTL/Th1 response induced by PC7A vaccine was abolished compared with wild type control. PC7A facilitates the cytosolic translocation of DNA, inducing cGAS-dependent STING activation.^17^ Further study showed that PC7A could also bind to STING directly and form biomolecular condensates to initiate the downstream type I IFN expression.^18^ This vaccine produced potent tumor growth inhibition in multiple tumor models and showed excellent synergy with checkpoint inhibitors.^17^

The route of vaccine administration affects performance and therapeutic outcome. Vaccines are traditionally introduced through muscle or skin. A previous study showed that incomplete Freund’s adjuvant, which is commonly used in clinical vaccine trials, could persist and attract T cells to injection sites, diverting them from tumors.^19^ This finding offers insights into why some vaccines were able to increase circulating tumor-specific T cells without significant tumor regression.^20 21^ In this study, we investigated the effects of two administration methods of the PC7A nanovaccine on the production and infiltration of antigen-specific cytotoxic T cells in tumor tissues and the resulting antitumor efficacy. We discovered that intratumoral (I.T.) delivery of the PC7A nanovaccine achieved significantly higher antitumor efficacy than subcutaneous (S.C.) injection with the same vaccine dose. Both components (ie, tumor antigens and PC7A polymer) are necessary for the efficacious antitumor response. Intratumoral delivery of the nanovaccine altered cytokine expression in myeloid cells, reinforced the CXCL9-CD8^+^ T/IFNγ feedback loop and reversed the immunosuppressive microenvironment, which led to a significantly higher number of infiltrating T cells for tumor eradication.

## Methods

### Preparation of PC7A nanovaccine

Micelles were prepared following a solvent evaporation method as previously published.^22^ After micelle formation, the nanoparticles were characterized by dynamic light scattering (Malvern MicroV model, He-Ne laser, λ=632 nm) to determine the hydrodynamic diameter (Dh). For nanovaccine preparation, antigenic peptide was dissolved in distilled water, mixed with PC7A nanoparticles and diluted with Phosphate Buffered Saline(PBS) to a final concentration of 10 µg of antigen peptide per mL or 300 µg of PC7A NP per ml.

### Mice

Wild-type C57BL/6 mice were purchased from GemPharmatech (Shanghai, China). STING^-/-^ mice were purchased from the Jackson Laboratory. All mice were maintained under specific pathogen-free conditions at 22–26 °C with a 12:12 h dark/light cycle and 40%–70% humidity. Wild-type female mice were used at an age of 6–8 weeks. For genetic-modified mice, age-matched and sex-matched mice were used for each experiment. All animal procedures were performed with ethical compliance and approval by the Institutional Animal Care and Use Committee at Fudan University.

### Immunization and antitumor efficacy studies

C57BL/6 mice (n=5–10 for each group) were injected subcutaneously with B16-OVA (1.5×10^5^), TC-1 cells (1.5×10^5^) or B16-F10 cells (1.5×10^5^) into the right flank. Animals were immunized by either subcutaneous injection of the nanovaccine at the tail base or intratumoral injection of nanovaccine (1 µg antigen peptide, PC7A NP 30 µg). Tumor growth was subsequently measured two times a week using a digital caliper and calculated as 0.5×length×width^2^. Mice were killed when the tumor volume reached 1500 mm^3^. For the cell depletion assay, mice were given 250 µg of anti-NK1.1 antibody, anti-CD8a antibody and anti-CD4 antibody four times by I.P. injection every 3 days per mouse during vaccination. For lung metastasis model, mice were injected subcutaneously with 1.5×10^5^ B16-OVA cells and intravenously with 1×10^5^ B16-OVA cells 4 days later. Lung metastasis was analyzed 20 days post intravenous injection. For FTY720 treatment, mice were intraperitoneally injected with 25 µg of FTY720 (Selleckchem) initially 1 day before vaccine treatment and were maintained every other day with 20 µg of FTY720 throughout the duration of the experiments.

### Lymph node imaging assay

To investigate whether NPs can accumulate in the draining LNs, we labeled the PC7A copolymer with indocyanine green (ICG, λ_ex_/λ_em_=800/820 nm). ICG-encoded PC7A NPs (30 µg per mouse) were injected subcutaneously at the tail base or intratumorally into TC-1 tumor xenografts (50–100 mm^3^) in C57BL/6 mice. NP distribution was imaged using a clinical camera (SPY Elite). Animals were sacrificed 24 hours after the injection of NP, and major organs and inguinal and axillary LNs were excised and imaged.

### In vivo cytotoxicity killing assay

One week after immunization, naïve C57BL/6 mice were sacrificed, and splenocytes were collected. Half of the splenocytes were pulsed with E7_49-57_ peptides for 2 hours in complete medium at 37°C. The unplused and peptide-pulsed cells were labeled with 0.5 or 0.05 µM carboxyfluorescein succinimidyl ester (CFSE), respectively. Equal numbers of CFSE^low^ (E7_49-57_ pulsed cells) and CFSE^high^ (unplused cells) were mixed together and injected intravenously into the immunized mice. After 16 hours, the blood from treated mice was collected and subjected to flow cytometry analysis. The number of CFSE^high^ and CFSE^low^ cells was determined and used to calculate the percentage of E7_49-57_ peptide-pulsed target cell killing. Specific killing was defined as the percentage of specific lysis=(1 − nontransferred control ratio/experimental ratio) ×100.

### Flow cytometry

Spleens were harvested under sterile conditions. Blood was harvested with heparin, and red blood cells were removed using RBC lysis buffer. Subcutaneous and tumor tissues were digested by 0.25 mg/mL collagenase IV (Sigma–Aldrich) and 0.2 mg/mL DNase I (Sigma–Aldrich) for 20 min at 37°C. The murine antibodies purified antimouse CD16/32 (clone: 93), PE-Cy7 antimouse B220 (clone: RA3-6B2), Pacific Blue antimouse/human CD11b (clone: M1/70), Brilliant Violet 510 antimouse CD11c (clone: N418), FITC antimouse CD3 (clone: 145–2 C11), APC-Cy7 antimouse CD4 (clone: GK1.5), BV510 antimouse CD45 (clone: 30-F11), APC antimouse CD45 (clone: 30-F11), APC antimouse CD49b (clone: DX5), PE antimouse CD69 (clone: H12F3), eFluor 450 antimouse CD8a (clone: 53–6.7), PE-Cy7 antimouse CD86 (clone: GL-1), FITC antimouse Ly-6C (clone: HK1.4), APC/Cy7 antimouse Ly-6G (clone: 1A8), APC antimouse Ly-6G/Ly-6C (Gr-1) (clone: RB6-8C5) and PE antimouse MHCII (clone: M5/114.152.2) were used for flow cytometry. Flow cytometry data were acquired on a BD LSR II flow cytometer and analyzed using FlowJo software.

### Multiplex chemokine array

Chemokine concentrations in tumor homogenates were measured by a Legendplex Mouse Proinflam Chemokine Panel (Biolegend, Cat# 740451). All procedures were performed according to the manufacturer’s instructions.

### Intracellular cytokine staining

Cells were subjected to intracellular cytokine staining with a staining buffer set (Invitrogen, Cat#:00–5523) according to the manufacturer’s instructions. For Foxp3 staining, cells were labeled with anti-CD45-BV510, anti-CD3-ef450, and anti-CD4-APC-Cy7 before membrane permeabilization and intracellularly labeled with Foxp3-AF488. For IFNγ staining, 5×10^5^ cells were incubated with 1 µg/mL E7_49-57_ peptide and 1 µg/mL brefeldin A for 6 hours before intracellular cytokine staining. For intracellular cytokine staining, cells were labeled with anti-CD45-BV510, anti-CD3-ef450, anti-CD4-APC-Cy7 and anti-CD8-APC before membrane permeabilization and later intracellularly labeled with anti-IFNγ-PE. For CXCL9 staining, 5×10^5^ cells were incubated with 1 µg/mL brefeldin A for 4 hours, and labeled with anti-CXCL9-PE.

### Macrophage differentiation

To obtain bone marrow derived macrophages (BMDMs), cells from bone marrow were collected, and red blood cells were removed using RBC lysis buffer. Cells were cultured with RPMI 1640 containing 10% fetal bovine serum(FBS) and 20 ng/mL M-CSF. Five to 7 days after M-CSF stimulation, cells were collected for experiments.

### Chemotaxis assay

To measure the chemotactic ability of CXCL9, splenocytes from TC-1 tumor-bearing mice were stained with anti-CD45-BV510, anti-CD3-FITC, anti-CD4-APC-Cy7, anti-CD8-ef450 and anti-CD49b-APC antibodies. To measure the chemotactic ability of PC7A-stimulated myeloid cells, CD3^+^ cells enriched by MACS were stained with anti-CD4-APC-Cy7 and anti-CD8-ef450 antibodies. A total of 600 µL of culture medium containing 900 ng/mL CXCL9 (Sino Biological, Cat# 50155-MNAE) or conditioned medium was loaded in the bottom chamber, and 5×10^5^ labeled cells were added to the top chamber. Migration was evaluated after 3–4 hours by quantification of the number of migrated cells in the bottom chamber using flow cytometry analysis. A 24-well Transwell system with 5 µm pores (BIOFIL) was used to measure the migration of splenocytes.

### Q-PCR

For tumor gene expression analysis, tumor tissues were lysed, and RNA was purified and reverse transcribed according to the manufacturer’s instructions. The following primers were used for qPCR.

mIFNγ: ATGAACGCTACACACTGCATC, CCATCCTTTTGCCAGTTCCTC; mCXCL9: TCCTTTTGGGCATCATCTTCC, TTTGTAGTGGATCGTGCCTCG; mIFNβ: ATGAGTGGTGGTTGCAGGC, TGACCTTTCAAATGCAGTAGATTCA; mTGFβ: CTCCCGTGGCTTCTAGTGC, GCCTTAGTTTGGACAGGATCTG mActin: ATGACCCAAGCCGAGAAGG, CGGCCAAGTCTTAGAGTTGTTG.

### Statistical analysis

Based on pilot immunization and tumor treatment studies, we used group sizes of 3–6 animals/group for immunogenicity measurements and 5–10 animals/group for tumor therapy experiments. Statistical analysis was performed using Microsoft Excel and Prism 5.0 (GraphPad). Data are expressed as mean±SEM. Data were analyzed by Student’s t-test. The variance similarity test (*f*-test) was performed before the *t*-test. All *t*-tests were one-tailed and unpaired and were considered statistically significant if p<0.05 (*p<0.05; **p<0.01; ***p<0.001; ****p<0.0001 unless otherwise indicated).

## Results

### I.T. administration of nanovaccine enhances antitumor efficacy over S.C. injection

**Figure 1:**
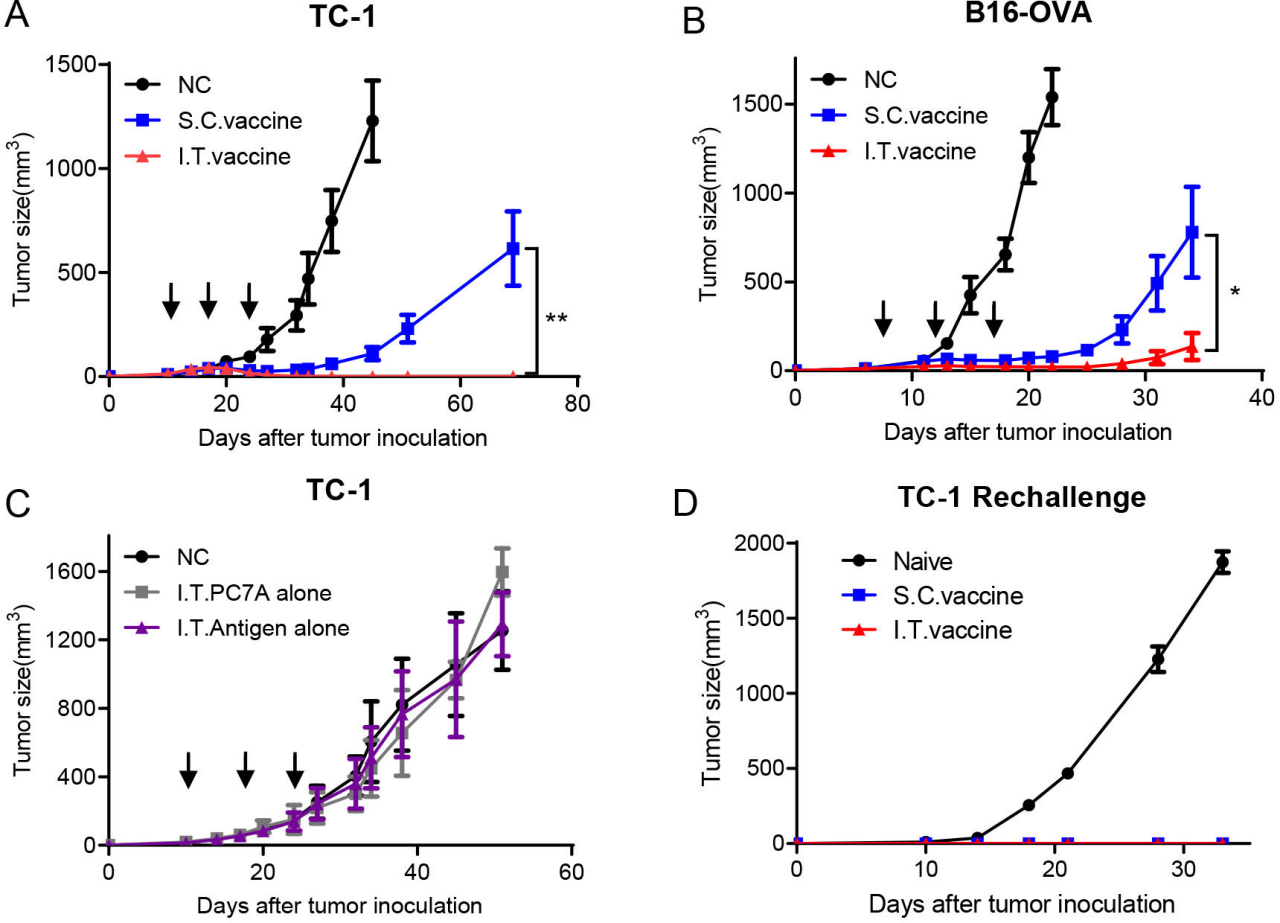
I.T. injection of nanovaccine enhances antitumor effects compared with S.C. injection and induces a long-term memory effect. (A) C57BL/6 mice were inoculated subcutaneously with TC-1 tumor cells (1.5×10^5^ per mouse) into the right flank. Mice received I.T. or S.C. vaccination (1 µg E7 peptide plus 30 µg PC7A polymer per mouse) on days 10, 17 and 24 after tumor inoculation, and I.T. injection of vehicle buffer PBS was included as negative control (n=10). (B) C57BL/6 mice were inoculated subcutaneously with B16-OVA tumor cells (1.5×10^5^ per mouse) into the right flank and were vaccinated I.T. or S.C. (1 μg OVA peptide plus 30 µg PC7A per mouse) on days 7, 12 and 17 after tumor inoculation (n=5). (C) TC-1 tumor-bearing mice were treated with PC7A NP or E7 antigen alone by I.T. injections, and I.T. PBS was included as negative control (n=5). (D) Naïve or tumor-free mice 60 days after tumor inoculation in the TC-1 model were challenged with 1× 10^6^ TC-1 tumor cells (n=5). Long-term memory effects were found in the two treated groups. **P<0.01, *p<0.05. I.T., intratumoral; S.C., subcutaneous.

HPV16 E7_43-62_-PC7A vaccines were administered intratumorally or subcutaneously to TC-1 tumor bearing mice when the tumors reached ~40 mm^3^ in size, followed by two additional boosting vaccinations every 7 days. Both delivery approaches inhibited tumor growth, while I.T. vaccination achieved a higher tumor regression response than S.C. vaccination (figure 1A). All mice in the I.T. group were found to be tumor free, whereas only two mice in the S.C. group had no tumor (online supplemental figure S1A, B). Enhanced antitumor efficacy by I.T. administration was also observed in the B16-OVA tumor model compared with S.C. injection (figure 1B and online supplemental figure S1C, D). Furthermore, with an antigen cocktail of tumor-associated antigens and neoantigens, I.T. injection of PC7A vaccines also produced a more efficient inhibition on the growth of B16-F10 tumor than the S.C. route (online supplemental figure S1E, F).

10.1136/jitc-2021-003960.supp1 Supplementary data

To evaluate the specific contribution of tumor antigens or the adjuvant effect of PC7A NPs on antitumor immunity, we investigated tumor growth inhibition by I.T. administration of either tumor antigens or PC7A NPs alone. Neither group showed a significant difference in tumor retardation from the no-treatment control (figure 1C), indicating that both components are necessary to achieve antitumor immunity. We also investigated the long-term immune memory effects using tumor-free mice from the I.T. and S.C. groups. Mice were rechallenged with 1×10^6^ TC-1 tumor cells, and no tumors were found in either group compared with the naïve mouse group (figure 1D). This result showed that I.T. vaccination can also induce a long-term memory response similar to S.C. vaccination as previously reported.^17^

### Antitumor effect of I.T. vaccination is dependent on CD8^+^ T cells

**Figure 2:**
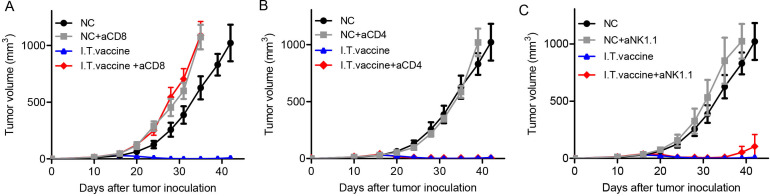
Antitumor effect of I.T. vaccination is dependent on CD8^+^ T cells. Mice were treated with the PC7A nanovaccine on days 10, 17 and 24. One day before vaccine treatment, 250 µg of depletion antibodies against CD8^+^ T cells (A), CD4^+^ T cells (B) or NK cells (C) were injected I.P. every 3 days for a total of four times(n=5). CD8^+^ T cells but not CD4^+^ T cells or NK cells are important for tumor eradication after I.T. vaccination. I.T. PBS was included as negative control in the experiments. I.T., intratumoral.

CD8^+^ T cells, CD4^+^ T cells and NK cells are the three main types of immune effector cells in cancer immunotherapy.^23 24^ We used a cell depletion assay to investigate which cell population plays a major role in antitumor immunity after I.T. vaccination. One day before vaccine treatment, antibodies (250 µg) blocking CD8^+^ T cells, CD4^+^ T cells or NK cells were administered four times by intraperitoneal (I.P.) injection every 3 days. CD8^+^ T cell depletion abolished most tumor retardation (figure 2A), while depletion of CD4^+^ T cells or NK cells did not affect tumor growth inhibition compared with the I.T. vaccine group (figure 2B, C). These results suggest that CD8^+^ T cells are primarily responsible for tumor eradication by I.T. vaccination.

### I.T. vaccination increases T cell accumulation in tumors

**Figure 3:**
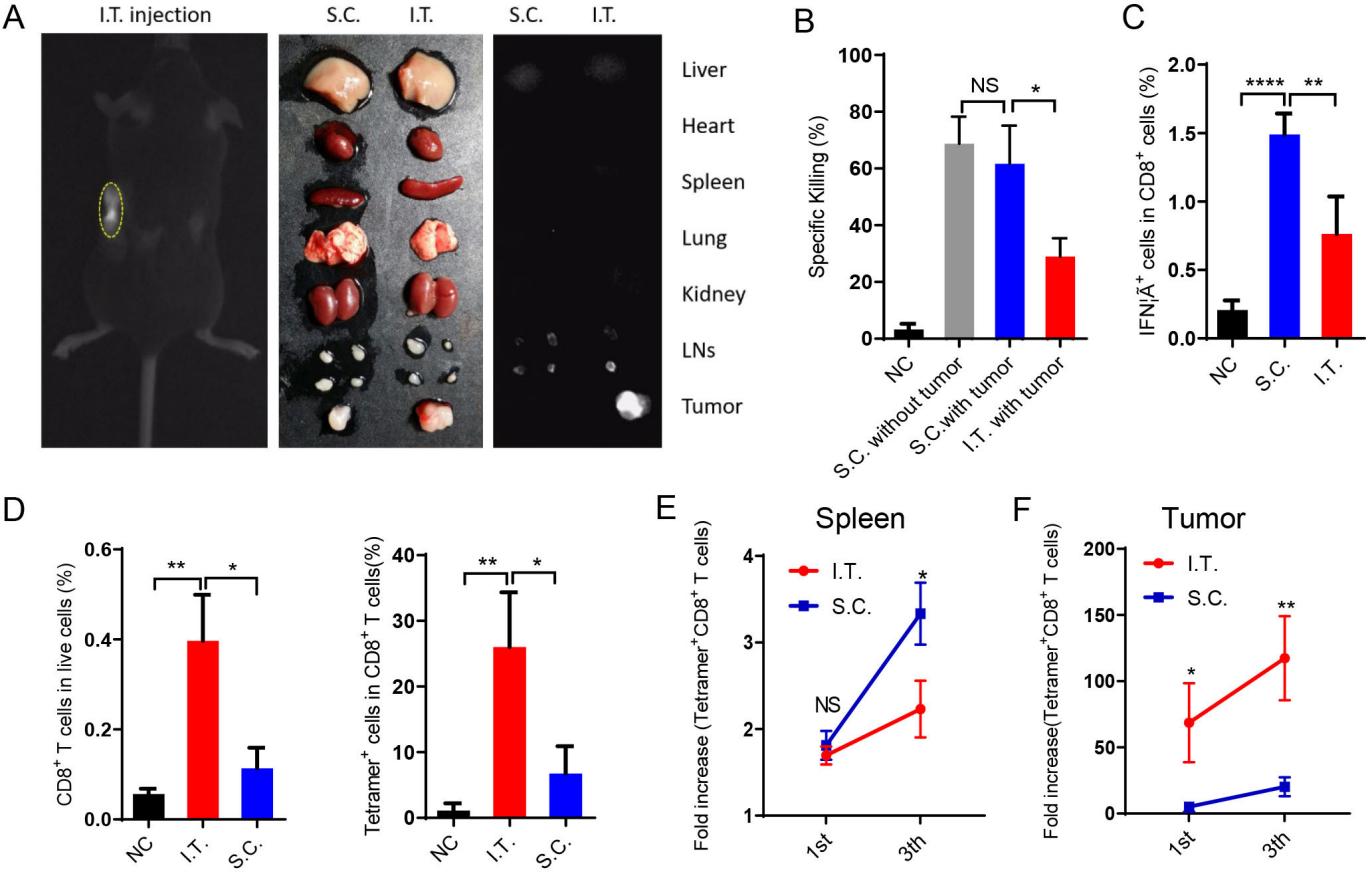
I.T. injection of nanovaccine increases antigen-specific T cell accumulation in tumors compared with S.C. injection. (A) Near-infrared imaging of ICG-labeled PC7A NP accumulation in tumors and tdLNs after I.T. injection. After 24 hours, in vivo imaging showed the most PC7A accumulation in tumors on the left side. (B) The systemic cytotoxic T cell response was detected by an in vivo CTL assay after the first immunization. (C) Splenocytes were stimulated with E7_49-57_ peptides and intracellularly stained for IFNγ after the third immunization. The proportion of IFNγ^+^ cells among CD8^+^ T cells was measured by flow cytometry. (D) Percentage of CD8^+^ T cells and tetramer^+^ CD8^+^ T cells in tumors after the first immunization. (E) Quantification of tetramer^+^CD8^+^ T cells in the spleen after the first and last vaccination. (F) Quantification of tetramer^+^CD8^+^ T cells in the tumors after the first and last vaccination (n=5). In panels B–D, I.T. PBS was included as negative control. ****P<0.0001, ***p<0.001, **p<0.01, *p<0.05. One-way ANOVA t-test. ANOVA, analysis of variance; I.T., intratumoral; NS, not significant; S.C., subcutaneous.

We then investigated the draining of nanovaccines in the LNs after I.T. or S.C. administration using ICG-labeled PC7A nanoparticles. The results showed that S.C. injection resulted in nanovaccine accumulation in LNs on both sides but none in the tumor. In the I.T. group, most labeled particles were trapped in tumors and tumor-draining LNs. The two ipsilateral LNs showed fluorescence and were enlarged, whereas the contralateral LNs showed normal size and minimal fluorescence (figure 3A). Other organs did not show significant accumulation in either group.

The T cell killing activities induced by immunization were measured in vivo. S.C. injection induced two-fold higher systemic antigen-specific T cell killing activity than I.T. injection (figure 3B). In LNs, S.C. injection showed superior activation of both antigen-presenting cells (DCs and macrophages) and lymphocytes (CD4^+^, CD8^+^, NK cells) (online supplemental figures S2 and S3A–D). These activations were dependent mainly on the STING pathway (online supplemental figures S3E, F). Antigen-specific T cells were also analyzed in vitro. After the third vaccination, spleen cells were dispersed into a single cell suspension and stimulated with E7_49-57_ peptides, and then the percentage of CD8^+^ IFNγ^+^ T cells (antigen-specific T cells) was measured by flow cytometry. In the S.C. injection group, ~1.5% of CD8^+^ T cells were IFNγ^+^, which was significantly higher than that in the I.T. injection group (figure 3C). These data demonstrated that S.C. administration of the PC7A nanovaccine generated a higher level of systemic T cell activity than I.T. injection.

In contrast, I.T. injection led to a higher number of CD8^+^ T cells inside the tumor than S.C. injection. In tumor tissues, antigen E7-specific CD8^+^ T cells comprised up to 26% of total CD8^+^ T cells after I.T. injection, compared with only 6.7% in the S.C. group (figure 3D). Consistently, although the proportion of tetramer^+^CD8^+^ T cells in the LNs and spleen was higher following S.C. vaccination than I.T. vaccination (figure 3E and online supplemental figures S3D), tetramer^+^CD8^+^ T cells were dramatically elevated within tumor tissues (69-fold and 117-fold in I.T. group over the control group after the first and final vaccination, respectively, compared with 5.1-fold and 20-fold in the S.C. group, figure 3F). Therefore, I.T. injection of the PC7A nanovaccine significantly increased the accumulation of antigen-specific T cells in tumor tissues.

### I.T. vaccination enhances CXCL9/IFNγ-correlated T cell recruitment

**Figure 4:**
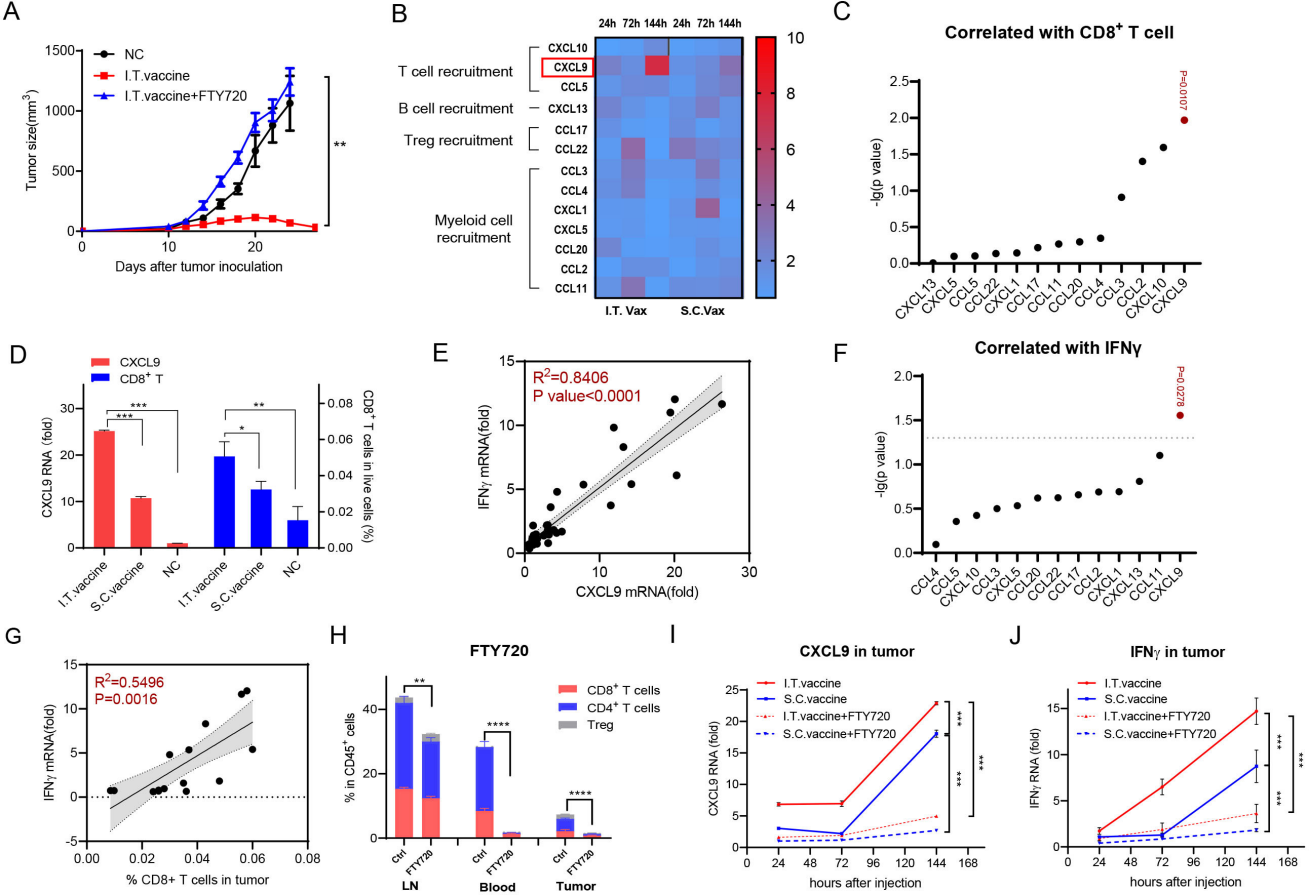
CD8^+^ T cell infiltration induced by I.T. injection correlates well with CXCL9 and IFNγ expression. (A) Tumor growth inhibition was analyzed with or without FTY720 treatment (n=5). (B) Quantification of chemokine expression at 24 hours, 72 hours and 144 hours after treatment. Fold increase of individual protein pictogram abundance per milligram of tumor tissue was shown (n=3). (C) Correlation of CD8^+^ T cells infiltration with different chemokines in tumor. (D) CXCL9 and CD8^+^ T cell level in tumor 144 hours after different treatment. (E) Correlation of IFNγ mRNA in tumor with CXCL 9 mRNA level in tumor. (F) Correlation of IFNγ mRNA in tumor with different chemokines in tumor. (G) Correlation of IFNγ mRNA in tumor with CD8^+^ T cell infiltration in tumor. (H) Quantification of T cell migration with or without FTY720 treatment. (I, J) Quantification of CXCL9 and IFNγ expression level in tumor with or without FTY720 treatment. In panel D, I.T. PBS was included as negative control. ****P<0.0001, ***p<0.001, **p<0.01, *p<0.05. One-way ANOVA t-test. ANOVA, analysis of variance; I.T., intratumoral; NS, not significant; S.C., subcutaneous.

The T cells in the tumor microenvironment either expand from pre-existing T cells inside the tumor itself or migrate from peripheral lymphoid tissues into tumor sites. To determine the origin of T cells that accumulated in tumors after I.T. vaccination, we used FTY720 to block peripheral lymphocyte circulation into tumor sites. Mice were intraperitoneally injected with FTY720 1 day before vaccine administration and were maintained every other day. I.T. vaccination failed to inhibit tumor growth after FTY720 treatment (figure 4A), indicating that lymphocyte trafficking from peripheral lymphoid tissues into tumors is crucial for tumor eradication.

Chemokines play critical roles in cell migration. To determine the mechanism of I.T. vaccination in T cell recruitment, we performed chemokine profiling in tumors on days 1, 3 and 6 after the first vaccination. CXCL9 (C-X-C motif chemokine ligand 9) continued to increase more than sevenfold by day 6 after I.T. vaccination, which was significantly higher than that after S.C. vaccination (figure 4B). Among the tested chemokines, CXCL9, which is able to regulate the migration of Th1 cells,^25^ showed the highest correlation with CD8^+^ T cell infiltration in tumors (figure 4C and online supplemental figure S4A). Compared with the normal compartments, the CXCL9 protein level in the tumor showed little difference but increased ~8 fold after I.T. vaccination (online supplemental figure S4B). Furthermore, in the I.T. group, the expression level of CXCL9 was significantly higher than that in the S.C. group, and the same pattern was observed in CD8^+^ T percentages in these tumors (figure 4D). CXCL10 and CCL2, regulating the recruitment of Th1 cells and myeloid cells^25 26^ respectively, were also found to be correlated with CD8^+^ T cell infiltration (figure 4C). However, their correlations were lower than CXCL9, and their protein levels changed little with vaccination (figure 4B, C, online supplemental figure S4B). Therefore, I.T. vaccination specifically induced higher expression of CXCL9, which was correlated with T cell recruitment, than S.C. vaccination.

The PC7A nanovaccine can activate IFNγ-expressing T lymphocytes efficiently (figure 3C), and CXCL9 is sensitive to IFNγ.^27^ To determine their relationships, we detected IFNγ expression in tumors. Matched scatterplots showed that the expression level of IFNγ was highly correlated with that of CXCL9 but not other chemokines (figure 4E, F). In addition to CXCL9, IFNγ showed a significant correlation with CD8^+^ T cell infiltration in tumors (figure 4G). Dynamic analysis showed that CXCL9 and IFNγ levels in tumors continued to increase after either I.T. or S.C. vaccination, and both were significantly higher in the I.T. group than in the S.C. group (figure 4I, J). When treated with FTY720, most T cells were sequestered in LNs (figure 4H), and the IFNγ and CXCL9 levels in tumors were also substantially decreased (figure 4I, J), indicating that vaccination-induced upregulation of IFNγ and CXCL9 is dependent on infiltrated lymphocytes. Taken together, all these data revealed that I.T. administration of the nanovaccine induced higher expression of IFNγ/CXCL9 and more T cell infiltration into tumors than S.C. vaccination.

### PC7A in I.T. vaccination initiates the myeloid cell/CXCL9-CD8^+^ T/IFNγ feedback loop for T cell recruitment

**Figure 5:**
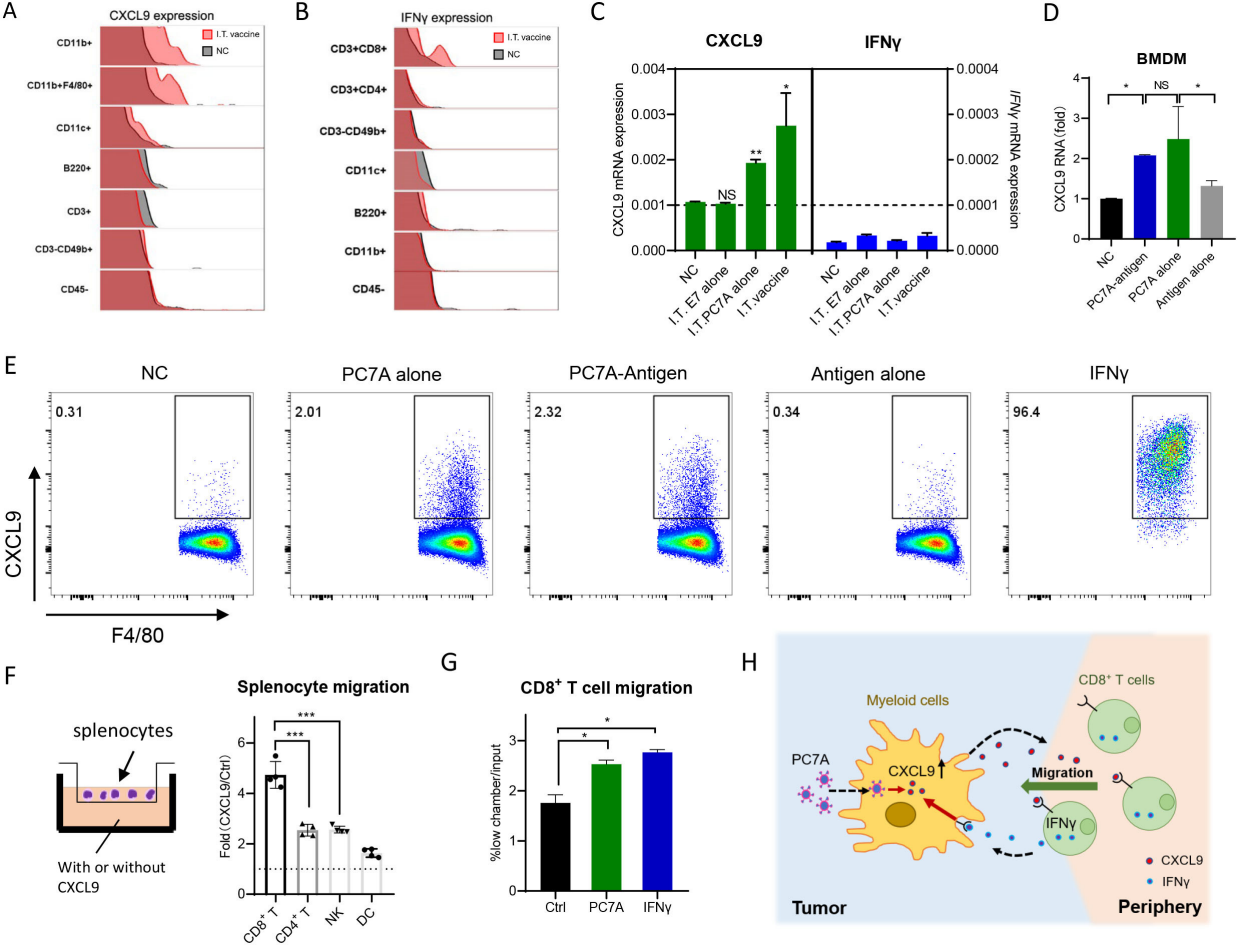
PC7A initiates myeloid cell/CXCL9-CD8^+^ T/IFNγ feedback loop for T cell recruitment. (A) CXCL9 expression in different cell subpopulations from TC-1 tumors were measured by intracellular staining 24 hours after I.T. vaccination. (B) IFNγ expression in different cell subpopulations from TC-1 tumors were measured by intracellular staining 6 days after I.T. vaccination.(C) Quantification of CXCL9 and IFNγ mRNA expression in TC-1 tumor derived CD45^+^ cells 24 hours after PC7A, antigen peptide or vaccine I.T. treatment (n=3). (D) Quantification of CXCL9 mRNA in BMDM stimulated with PC7A-antigen or PC7A alone, antigen alone for 24 hours. (E) Intracellular staining of CXCL9^+^ cells in peritoneal macrophage stimulated with PC7A alone, antigen alone, PC7A-Antigen and IFNγ for 12 hours. (F) Chemotaxis assay of splenocytes derived from tumor bearing mice toward media with or without 900 ng/mL CXCL9. Migrated CD4 ^+^ T cells, CD8 ^+^ T cells, NK were quantified by flow cytometry. (G) BMDMs in the lower chamber were treatment with 100 µg/mL PC7A or 1 ng/mL IFNγ for 8 hours, chemotaxis assay of splenocytes derived from tumor bearing mice was quantified by flow cytometry. (H) Schematic of myeloid cell/CXCL9-CD8^+^ T/IFNγ and the effect of PC7A. In panels A–E, I.T. PBS was included as negative control. ****P<0.0001, ***p<0.001, **p<0.01, *p<0.05. One-way ANOVA t-test. ANOVA, analysis of variance; I.T., BMDM, bone marrow-derived macrophage; I.T., intratumoral; NS, not significant.

To examine the interplay of CXCL9, IFNγ and T cell infiltration, we first performed intracellular staining to determine the cellular sources of upregulated CXCL9 and IFNγ in tumors after I.T. vaccination. CXCL9 was upregulated 24 hours after vaccination in myeloid cells, especially macrophages, but not lymphocytes or tumor cells (figure 5A and online supplemental figure S4C). Upregulation of IFNγ was clearly detected in CD8^+^ T cells at day 6 after vaccination (figure 5B and online supplemental figure S4C). To identify which component of the nanovaccine contributes to this effect, tumor-bearing mice were treated with PC7A or E7 antigen peptide alone intratumorally for 24 hours. CXCL9 was significantly upregulated by both nanovaccine alone and PC7A alone but not antigen alone, and IFNγ expression displayed little difference among groups (figure 5C). Furthermore, both the nanovaccine alone and PC7A alone, but not E7 alone, induced the upregulation of CXCL9 in BMDMs or peritoneal macrophages in vitro (figure 5D, E), indicating that I.T. vaccination stimulates CXCL9 expression in myeloid cells through the adjuvant PC7A.

To test CXCL9-induced T cell recruitment, a chemotaxis assay of splenocytes derived from tumor-bearing mice showed that CD8^+^ T cells were the major cell population that migrated in response to CXCL9 (figure 5F), indicating that CXCL9 can stimulate the recruitment of IFNγ-expressing CD8^+^ T cells. Furthermore, IFNγ induced substantial upregulation of CXCL9 in macrophages (figure 5E), and conditioned medium from BMDMs treated with either PC7A or IFNγ significantly increased CD8^+^ T cell migration (figure 5G). These results demonstrated that I.T. injection of the PC7A nanovaccine initially stimulates CXCL9 expression in myeloid cells, recruiting CD8^+^ T cells to produce IFNγ, which reciprocally activates myeloid cells to secrete more CXCL9, forming a myeloid cell/CXCL9-CD8^+^ T/IFNγ feedback loop for enhanced T cell recruitment (figure 5H).

### I.T. vaccination initiates CXCL9 expression through STING pathway

**Figure 6:**
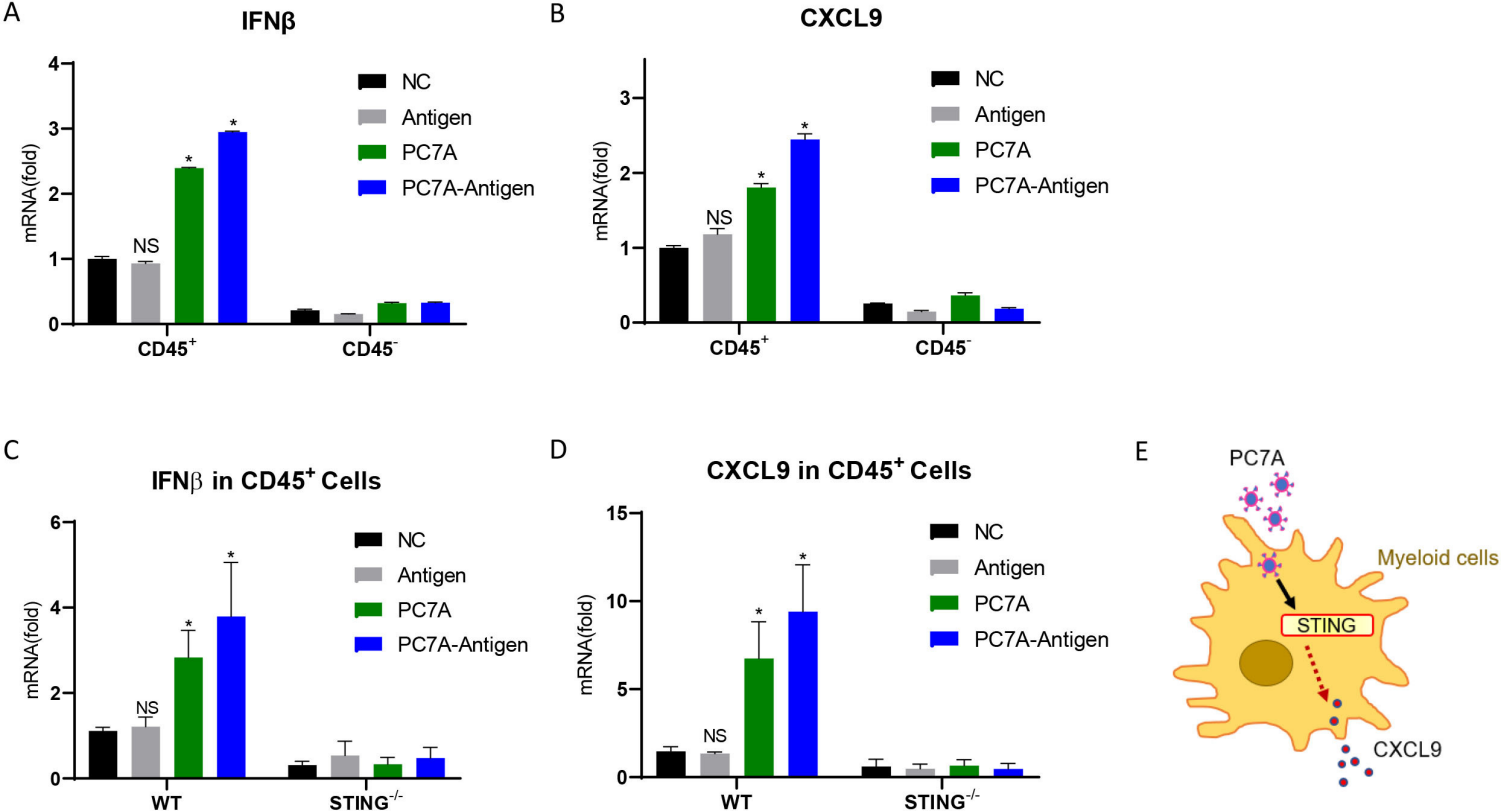
PC7A initiates CXCL9 expression through STING pathway. (A) Quantification of IFNβ expression in tumor derived CD45^+^ cells and CD45^-^ cells 24 hours after I.T. treatment of PBS (NC), PC7A, antigen peptide or vaccine (n=3). (B) Quantification of CXCL9 expression in tumor derived CD45^+^ cells and CD45^-^ cells 24 hours after PC7A, antigen peptide or vaccine I.T. treatment (n=3). (C) Quantification of IFNβ expression in WT and STING^-/-^ mice 24 hours after I.T. treatment (n=4). (D) Quantification of CXCL9 mRNA expression in WT and STING^-/-^ mice 24 hours after PC7A, antigen peptide or vaccine I.T. treatment (n=4). (E) Schematic of STING dependent PC7A induced CXCL9 expression. *P<0.05. One-way ANOVA t-test. ANOVA, analysis of variance; I.T., intratumoral; NS, not significant.

Since PC7A could activate the STING-type I IFN pathway,^17^ we examined whether STING activation is required for PC7A to induce CXCL9 in tumor. The expression of IFNβ, the marker of STING activation, in tumors was measured 24 hours after I.T. vaccination. Result showed that it was significantly upregulated in tumor derived leukocytes (CD45^+^) but not tumor cells (CD45^-^), so did with the treatment of PC7A alone but not antigen alone (figure 6A). CXCL9 displayed similar expression pattern after these treatments (figure 6B). Hence, PC7A in nanovaccine activates STING pathway and CXCL9 in tumor derived leukocytes but not tumor cells. Then, we compared the expression of IFNβ and CXCL9 in tumor derived leukocytes from WT and STING^-/-^ mice. As expected, PC7A and nanovaccine cannot induce IFNβ upregulation in STING^-/-^ mice (figure 6C). Compared with WT mice, the expression of CXCL9 in tumor derived leukocytes from STING^-/-^ mice was also not altered by the I.T. treatment of either nanovaccine or PC7A alone (figure 6D). Therefore, these results indicated that PC7A-induced CXCL9 upregulation is dependent on the STING pathway (figure 6E).

We then compared the I.T. injection of PC7A nanovaccine with a STING agonist, Cyclic GMP-AMPP(cGAMP)^28^ in tumor-bearing mice. Multiple chemokines showed stronger upregulation by cGAMP than that achieved by vaccination, including chemokines involved in T cell infiltration and in the recruitment of Tregs and myeloid cells (online supplemental figure S5A). IFNβ showed a higher expression 24 hours after cGAMP injection compared with vaccination, consistent with our previous report^17^ and then dropped to normal level on day 6 (online supplemental figure S5B). Importantly, CXCL9 expression continued to increase from day 1 to 6 postvaccination; however, after cGAMP treatment, it increased during the first 3 days and then decreased substantially (online supplemental figure S5A, C). And IFNγ expression displayed the same pattern as CXCL9 after vaccination but was low from day 1 to day 6 after cGAMP treatment (online supplemental figure S5D). Cell profiling showed that both I.T. cGAMP and I.T. vaccination caused inflammation in the tumor microenvironment. cGAMP induced myeloid-biased infiltration, whereas vaccine initiated more lymphocyte and DC engraftments (online supplemental figure S5E). In LNs, I.T. cGAMP induced fewer tumor-specific CD8^+^ T cells than the vaccine (online supplemental figure S5F). The antitumor efficacies of the I.T. treatments were determined in TC-1 tumor bearing mice. PC7A alone has little effect, while cGAMP exhibited a minor degree of immune protection, and the nanovaccine showed the most substantial inhibition on tumor growth (online supplemental figure S5F). These results demonstrated that the PC7A nanovaccine initiates CXCL9 upregulation in a STING-dependent manner, but STING agonist (cGAMP) alone cannot support a sustained increase in CXCL9 and tumor-specific T cells without efficient antigen presentation, and both are required to establish the feedback loop of myeloid cell/CXCL9-CD8^+^ T/IFNγ in tumors.

### I.T. vaccination improves immune profiles in the tumor microenvironment

We investigated the immune profiles of the tumor microenvironment by the two vaccination methods. I.T. injection led to increased leukocytes (CD45^+^) in tumors (online supplemental figure S6A). For lymphoid cells, NK cells showed 1.9-fold increase compared with S.C. group. T cell activity was detected by the percentage of CD69^+^CD8^+^ and CD69^+^CD4^+^ T cells, which were 1.8-fold and 1.6-fold higher in the I.T. group over S.C. group (online supplemental figure S6B). For myeloid cells in tumors, tumor-associated macrophages (TAMs) were markedly decreased in the I.T. group. Furthermore, subtyping of TAMs showed that M1/M2 ratio increased by 1.8-fold in the I.T. group compared with S.C. group (online supplemental figure S6C). Additionally, the ratio of CD8^+^ T cells to regulatory T Cells (Tregs) correlates with positive clinical outcome,^29^ and TGFβ in tumor promotes cancer progression and represses the antitumor immunity.^30^ In our analysis, the ratio of CD8^+^ T /Tregs and the expression of TGFβ showed significant increase and decrease respectively in I.T. group when compared with control and S.C. group (online supplemental figure S6D, E). These results demonstrated that I.T. injection of PC7A nanovaccine inflamed the tumor microenvironment with an improved antitumor immunity over S.C. treatment.

### S.C. vaccination attracts; T cells to the subcutaneous injection site

We also investigated the STING mediated CXCL9 expression at S.C. injection site. The expression of IFNβ and CXCL9 was both markedly increased at injection site 24 hours after vaccination when compared with control treatment or noninjection site; however, the levels were then decreased; so did the percentage of CXCL9^+^ myeloid cells in these sites (online supplemental figure S7A–C), indicating inefficient myeloid cell/CXCL9-CD8^+^ T cell/IFNγ feedback loop formation. Polymer nanoparticles diffuse efficiently from injection site and accumulate in LNs.^31^ In the absence of tumor cells at S.C. injection site, diffusion of nanovaccine would result in diminished antigen stimulation on the recruited T cells, leading to the ablation of myeloid cell/CXCL9-CD8^+^ T cell/IFNγ loop over time. Even though, the expression level of CXCL9 and IFNγ was still significantly higher in S.C. injection sites than controls 144 hours after vaccination (online supplemental figure S7C), suggesting an accumulation of T cells. We then analyzed the accumulation of CD8^+^ T cells at the injection site and noninjection site in the S.C. group, as well as corresponding subcutaneous tissues of the I.T. and control groups after three vaccinations. The proportion of CD8^+^ T cells in the total leukocyte (CD45^+^) population was 3.8-fold higher at the injection site of S.C. group than in corresponding tissue from the I.T. group. Within the CD8^+^ T cell population, antigen-specific CD8^+^ T cells were 2.3-fold higher in the S.C. group than in the I.T. group. The percentage of tetramer^+^CD8^+^ T cells among total live cells from subcutaneous tissues was even further elevated, with a~13 fold increase for S.C. compared with I.T. vaccination (online supplemental figure S7D–E). These results illustrated that I.T. vaccination avoided T cell diversion outside tumors compared with S.C. injection.

**Figure 7:**
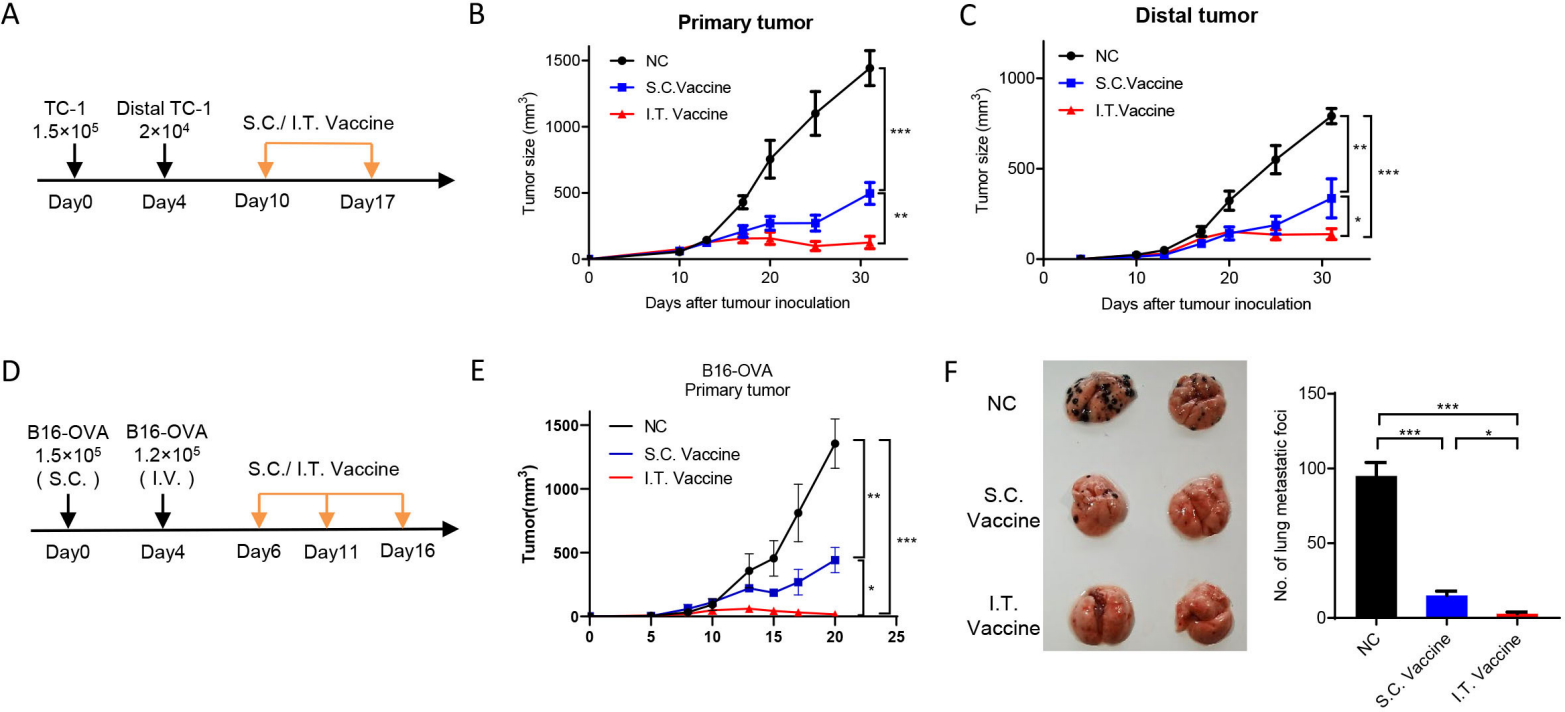
I.T. administration of nanovaccine shows higher distant antitumor efficacy. (A–C) C57BL/6 mice were inoculated with 1.5×10^5^ TC-1 cells on day 0 and 2×10^4^ TC-1 cells in the left flank on day 4. Mice were treated with I.T. or S.C. vaccination on days 10 and 17 and I.T. PBS was set as control (A). Tumor growth was monitored (n=5) (B, C). (D–F) C57BL/6 mice were subcutaneously inoculated with 1.5×10^5^ B16-OVA cells on day 0 and intravenously injected with 1×10^5^ B16-OVA cells on day 5. Mice received vaccination on days 6, 11 and 16 and I.T. PBS was set as control (n=5). (D) Growth curve of primary tumor (E) and the lung metastasis (F) were shown. ***P<0.001, **p<0.01, *p<0.05, One-way ANOVA t-test. ANOVA, analysis of variance; I.T., intratumoral; NS, not significant; S.C., subcutaneous.

### I.T. administration of nanovaccine enhances distant antitumor efficacy

To evaluate the antitumor efficacies of I.T. and S.C. vaccination on distal side, mouse models with bilateral tumors or lung metastasis were used. We first measured the abscopal effect. Primary tumors were first initiated by subcutaneous injection of TC-1 cells on the left flanks of mice, and distal tumors were introduced on the right flanks 4 days later. Mice received I.T. or S.C. vaccination at ~day 10 when the primary tumor reaches ~40 mm^3^ and the distal forms a palpable tumor (figure 7A). The results showed that both I.T. and S.C treatment efficiently inhibited the tumor growth on both sides when compared with control treatment, and I.T. vaccination achieved longer and more efficient inhibition than S.C. group (figure 7B, C). We further determined the vaccination efficacy on lung metastasis. Primary tumors were inoculated by subcutaneous injection of B16-OVA cells on the flanks of mice, and 4 days later, B16-OVA cells were injected intravenously for lung metastasis (figure 7D). After three dosages of vaccination, I.T. vaccination resulted in more efficient inhibition on both the growth of primary tumor and the lung metastasis compared with S.C. treatment (figure 7E, F).

## Discussion

Immune-activating nanoparticles have undergone rapid advances in the fields of biomaterials, nanomedicine and bioengineering.^32–35^ Most current applications have focused on systemic administration, such as subcutaneous or intravenous injections, and the resulting impact on immune activation.^36^ For cancer immunotherapy, the intratumoral injection of oncolytic virus or innate immune stimulators has shown therapeutic potential against tumor progression.^37 38^ Local injection of mRNA drugs such as mRNA-2416 delivered by lipid nanoparticles has been in a phase II clinical trial (NCT03323398). To date, few reports have systematically investigated the impact of administration methods on the performance of cancer vaccines.

We previously showed that the PC7A nanovaccine drained to LNs for T cell activation after subcutaneous injection. In this study, we demonstrated that intratumoral injection maintained LN targeting of nanovaccines to generate tumor-specific T cells and induced intratumoral CXCL9 expression to initiate a myeloid cell/CXCL9-CD8^+^ T cell/IFNγ feedback loop for CD8^+^ T cell infiltration, leading to a stronger antitumor effect and long-term immune memory than conventional subcutaneous immunization. Similar to the PC7A vaccine, the new generation of cancer vaccines mostly incorporate innate immune stimulators and tumor antigens, including neoantigens and nanomaterials, which show clinical potential.^8–11^ They were mainly introduced through the skin and sometimes LNs. Intratumoral administration suggested ways to further potentiate cancer vaccines for inoperable tumors and resection sites after surgery.^39^

To date, only a subset of patients benefit from existing cancer immunotherapy, and it is critical to identify precise and accurate predictive biomarkers for cancer therapeutic response, which would optimize the personalization of immunotherapy.^40^ A growing body of evidence suggests that CXCL9 in tumors regulates the recruitment of CXCR3-expressing stem-like CD8 T (Tstem) cells that underlie clinical responses to anti-PD(L)-1 treatment.^27^ Although CXCL9/CXCL10/CXCL11 all bind to CXCR3 expressed on CD8^+^ T cells, Th1 cells and NK cells, resulting in their migration to tumors, CXCL9 showed a stronger relationship with T cell infiltration and stronger antitumor immunity in human and mouse models.^41 42^ In our results, CXCL9 showed better sensitivity and specificity to vaccine stimulation and a stronger correlation with T cell infiltration and antitumor effects than CXCL10. The high CXCL10 expression in nontreated tumors implied that CXCL10 may have functions other than T cell recruitment, which could be the opposite.^43^ Hence, CXCL9 emerged as a potential biomarker of tumor immune recognition and T cell engraftment, which need further clinical verification.

Intratumoral injection of innate immune agonists has attracted much attention in cancer immunotherapy. Recently, intratumoral injection of TLR9 agonist, tilsotolimod, plus systemic delivery of ipilimumab did not result in improvement in objective response rate (ORR) over ipilimumab alone in a phase III melanoma trial (ORR of 8.8% vs 8.6%, NCT03445533).^44^ Multiple STING agonists are under clinical tests such as ADU-S100, MK-1454, GSK3745417 and so on, while single-agent human results have not been as promising.^45^ Our study revealed that I.T. PC7A nanovaccine initiated CXCL9 expression in myeloid cells in a STING dependent manner, recruiting CD8^+^ T cells to produce IFNγ, which reciprocally activated myeloid cells to secrete more CXCL9, forming a feedback loop for enhanced T cell recruitment. Although intratumoral injection of cGAMP similarly induced upregulation of CXCL9, other chemokines associated with the recruitment of myeloid cells and Tregs were also elevated. In addition, the intratumoral injection of cGAMP alone was less efficient in T cell priming to produce sufficient IFNγ compared with I.T. vaccination and cannot form a sustained myeloid cell/CXCL9-CD8^+^ T cell/IFNγ feedback loop. These results suggested that activation of the innate immune pathway and subsequent hyperproduction of broad-spectrum cytokines by these agonists can be a two-edged sword, which may be one reason for the lower-than-expected results of clinical trials.^46 47^ The intratumoral administration of nanoparticle-formulated drugs can enhance both retention and uptake by antigen-presenting cells, which would decrease the dose and augment the therapeutic effect for future design of STING agonist based immunotherapy.^48^

Our data show that subcutaneous injection of the nanovaccine induced higher levels of antigen-specific CD8^+^ T cells in systemic circulation than intratumoral injection. It is conceivable that the combination of subcutaneous and intratumoral administration of nanovaccines may be beneficial to produce synergistic antitumor immunity, particularly in immune-evasive cancers. Kudo-Saito *et al* reported that subcutaneous priming and intratumoral boosting with a recombinant poxvirus vaccine improved immune efficacy in advanced MC38 tumor models.^49^ Fujita *et al* found that intratumoral priming and subcutaneous boosting with an inactivated Sendai virus showed positive effects in patients with castration-resistant prostate cancer.^50^ Further investigation comparing the clinical efficacy of intratumoral administration versus the combination of subcutaneous and intratumoral administration of cancer nanovaccine will be necessary to generate preclinical proof of concept for clinical translation.

In summary, intratumoral administration of PC7A nanovaccine achieved stronger antitumor immunity and efficacy over subcutaneous injection. Mechanistic investigation showed STING mediated myeloid/CXCL9-CD8^+^ T/IFNγ feedback loop after intratumoral vaccination, which led to increased infiltration of tumor-specific cytotoxic T cells for tumor eradication. Our results indicate that intratumoral nanovaccine administration offers an efficient and efficacious approach to potentiate a new generation of cancer vaccines.

10.1136/jitc-2021-003960.supp2 Supplementary data

## Data Availability

Data are available on reasonable request.
