# Supplementary material for: Intratumoral administration of STING-activating nanovaccine enhances T cell immunotherapy
Source: J Immunother Cancer. 2022 May 26;10(5):e003960. doi: 10.1136/jitc-2021-003960 (PMC9150169; doi:10.1136/jitc-2021-003960)
Supplement: Supplementary data [file jitc-2021-003960supp001.pdf]

Supplementary Materials

Intratumoral Administration of STING-Activating Nanovaccine Enhances T Cell Immunotherapy

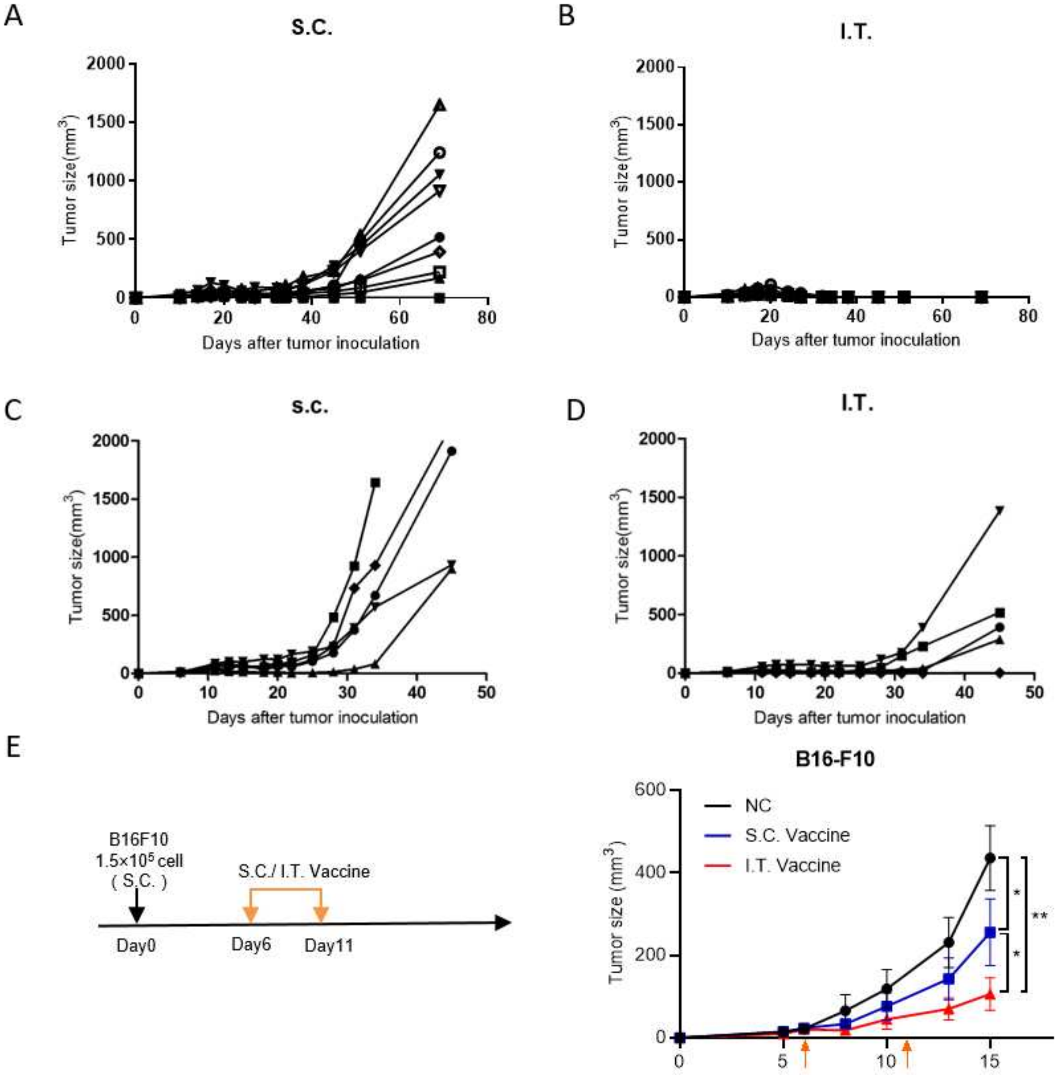

Supplementary Figure 1

Jiang X, *et al. J Immunother Cancer* 2022; 10:e003960. doi: 10.1136/jitc-2021-003960

Intratumoral injection of nanovaccine potentiates anti-tumor immunity compared to S.C. injection. (A-B) Single mouse growth curve of I.T. and S.C. in TC-1 model in main Figure 1A&B. C57BL/6 mice were transplanted S.C. with TC-1 tumor cells into the right flank. Mice were vaccinated i.t. or s.c. (1μg E7 peptide plus 30μg PC7A per mouse) for 3 times. The tumor growth curves were shown（n=10）. (C-D) Single mouse growth curve of I.T. and S.C. in B16-OVA model in main Figure 1C&D. C57BL/6 mice were transplanted S.C. with B16-OVA tumor cells into the right flank. Mice were vaccinated i.t. or s.c. (1μg OVA peptide plus 30μg PC7A per mouse) for 3 times. The tumor growth curves were shown（n=5）. (E-F) Schematic design and tumor growth curves of I.T. and S.C. injection of nanovaccine in B16-F10 model. Mice were inoculated subcutaneously with B16-F10 tumor cells (1.5×10^5 per mouse) and received I.T., S.C. vaccination (1.5μg peptide including $Gp100_{17–41}$, $Trp1_{214–237}$, $Obsl1_{T1764M}$, Pbk_{V145D}, $Tnpo3_{G504A}$, plus 30μg PC7A per mouse) or PBS on Days 6 and 11 after tumor inoculation（n=5）. **P<0.01, *P<0.05. NS, not significant, one-way ANOVA t-test.

Jiang X, *et al. J Immunother Cancer* 2022; 10:e003960. doi: 10.1136/jitc-2021-003960

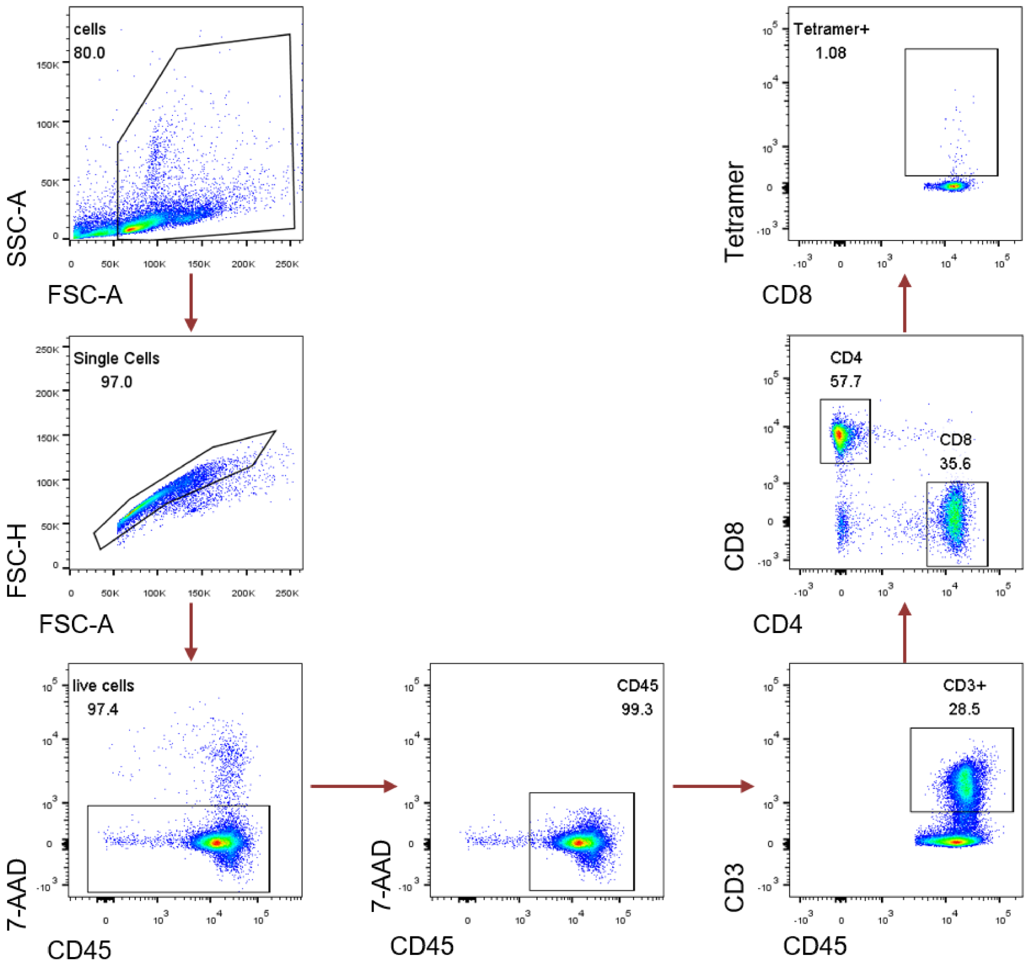

Supplementary Figure 2

Characterization of antigen-specific T cells by flow cytometry.

An example of the gating strategy used to define $CD4^+$ and $CD8^+$ T lymphocyte subsets. Viable lymphocytes were gated according to size, granularity and expression of CD3, CD4 and CD8. T cell subsets were classified as $CD4^+$ T cells ($CD45^+CD3^+CD4^+$) and $CD8^+$ T cells ($CD45^+CD3^+CD8^+$). The antigen specificity of $CD8^+$ T cells was identified by the H-2kb /SIINFEKL tetramer in TC-1 tumor models as $tetramer^+CD8^+$ T cells ($CD45^+CD3^+CD8^+tetramer^+$).

Jiang X, *et al. J Immunother Cancer* 2022; 10:e003960. doi: 10.1136/jitc-2021-003960

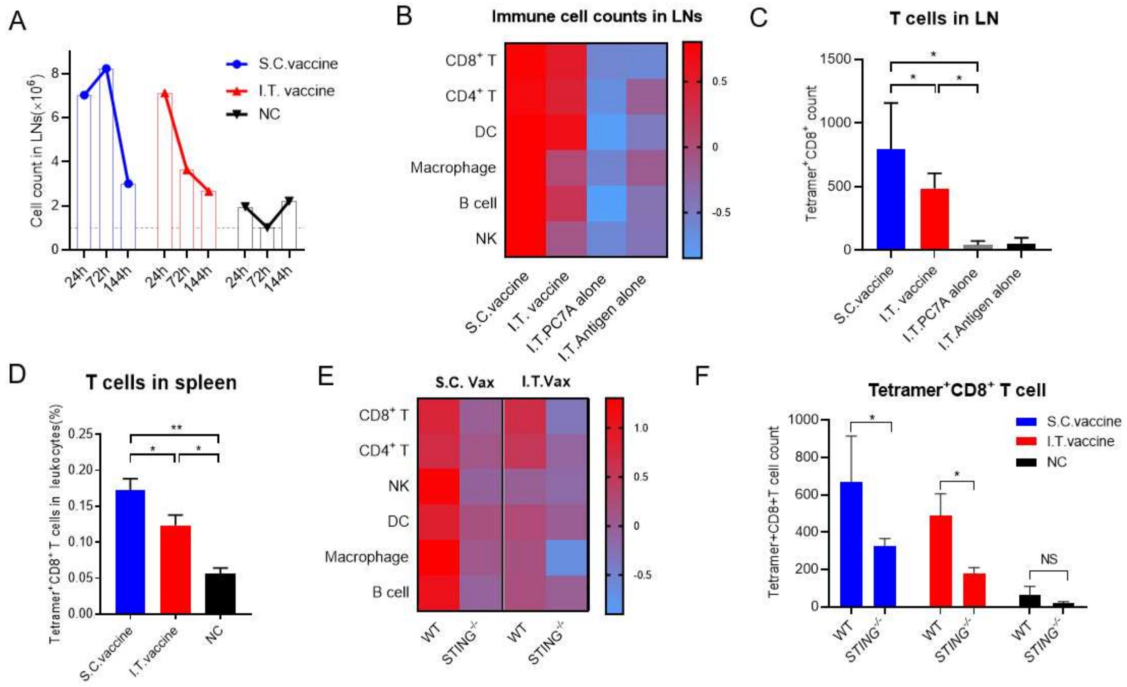

Supplementary Figure 3

I.T. injection of nanovaccine initiates peripheral T cell activation in a STING-dependent manner.

(A) Quantification of total cell numbers in lymph nodes at 24 h, 72 h and 144 h after the first treatment. (B) Quantification of different cell subpopulations 144 h after the first treatment. (C) Quantification of tetramer$^+$CD8$^+$ T cells 144 h after the first treatment in LNs. (D) Tetramer$^+$CD8$^+$ T cells in the spleen after the third vaccination. (E) Quantification of different cell subpopulations in the LNs in wild-type and *STING*$^{-/-}$ mice 144 h after the first treatment. (F) Quantification of tetramer$^+$CD8$^+$ T cells 144 h after treatment in LNs in wild-type and *STING*$^{-/-}$ mice. **P<0.01, *P<0.05. NS, not significant, one-way ANOVA t-test.

Jiang X, *et al. J Immunother Cancer* 2022; 10:e003960. doi: 10.1136/jitc-2021-003960

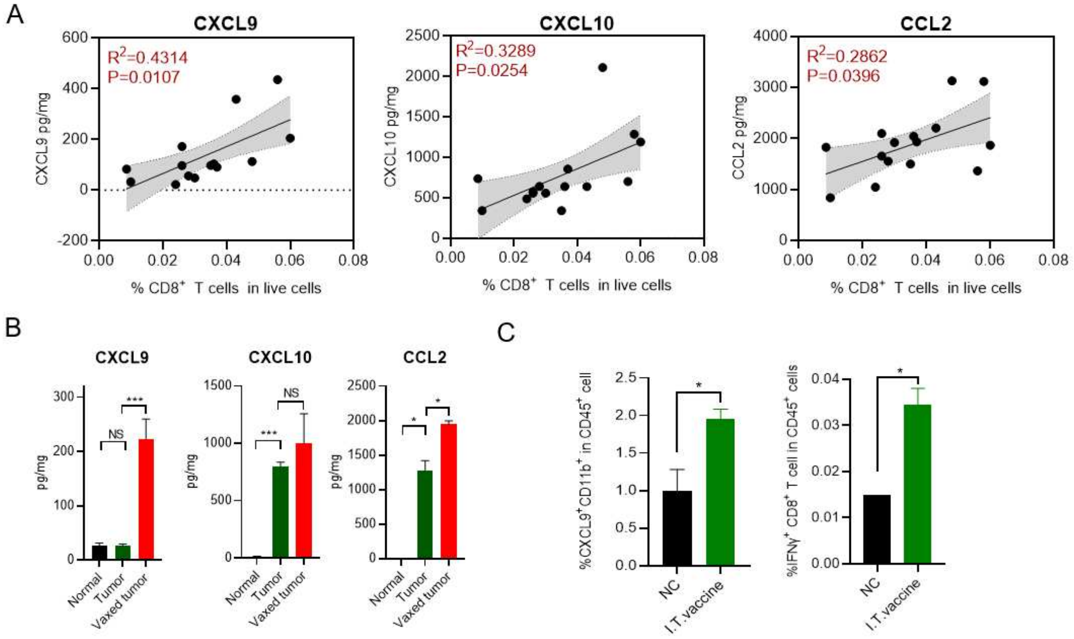

Supplementary Figure 4

CXCL9 upregulation in tumors is initiated by PC7A and further promoted by IFNγ from infiltrated T cells.

(A) Correlation of CXCL9, CXCL10 and CCL2 protein levels in tumors with the percentage of $CD8^+$ T cells in tumors. (B) Chemokine levels in normal subcutaneous tissues and tumor with or without I.T. vaccination. (C) Percentage of $CXCL9^+CD11b^+$cells and IFNγ $^+CD8^+$T cells in leukocytes in tumor 6 days after I.T.vaccination. ***P<0.001, **P<0.01, *P<0.05. NS, not significant, one-way ANOVA t-test.

Jiang X, *et al. J Immunother Cancer* 2022; 10:e003960. doi: 10.1136/jitc-2021-003960

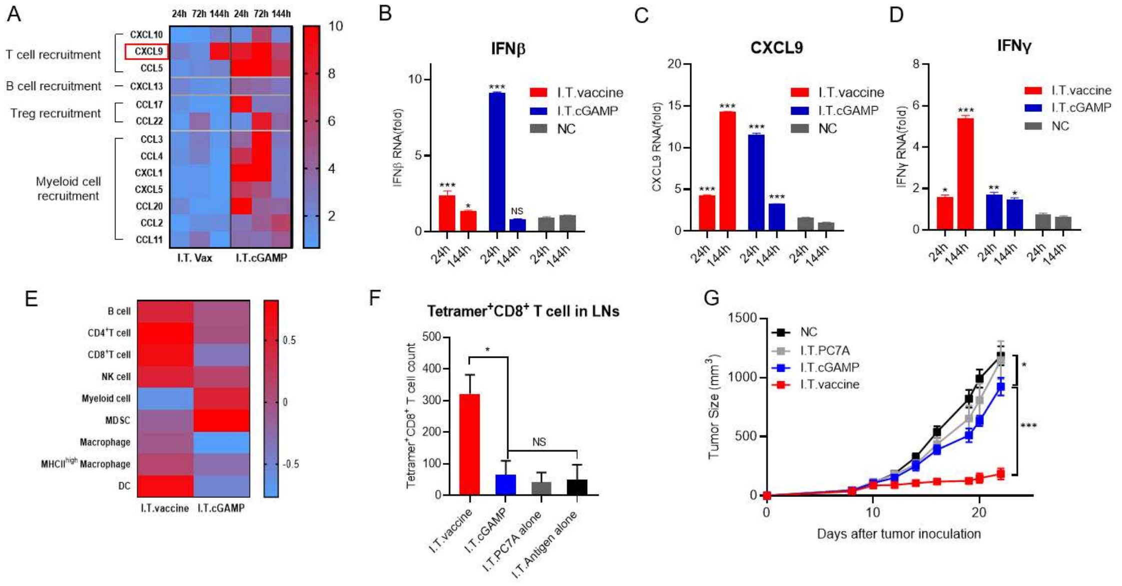

Supplementary Figure 5

cGAMP I.T. injection induces strong multiple cytokine expression but cannot sustain myeloid cell/CXCL9-CD8+ T/IFNγ feedback loop without T cell activation in periphery. (A) Tumor bearing mice were i.t. treated with 2ug cGAMP or vaccine (1μg E7 peptide plus 30μg PC7A per mouse). Quantification of chemokine expression was measured at 24 h, 72h and 144h after treatment. Fold increase of individual protein pictogram abundance per milligram of tumor tissue was shown. (B-D) Quantification of IFNβ, CXCL9 and IFNγ mRNA expression at 24hours and 144hours after treatment. (E) Quantification of different cell subpopulations in leukocytes 6days after treatment. (F) Quantification of Tetramer+ $CD8^{+}$ T cells 6 days after treatment. (G) TC-1 tumor bearing mice were i.t. treated with PC7A (50μg per mouse) or cGAMP (2μg per mouse) on day 10/13/16, or vaccine on day 10/17 after tumor inoculation. Tumor growth inhibition were analyzed after tumor inoculation. ***P<0.001, **P<0.01, *P<0.05. NS, not significant, one-way ANOVA t-test.

Jiang X, *et al. J Immunother Cancer* 2022; 10:e003960. doi: 10.1136/jitc-2021-003960

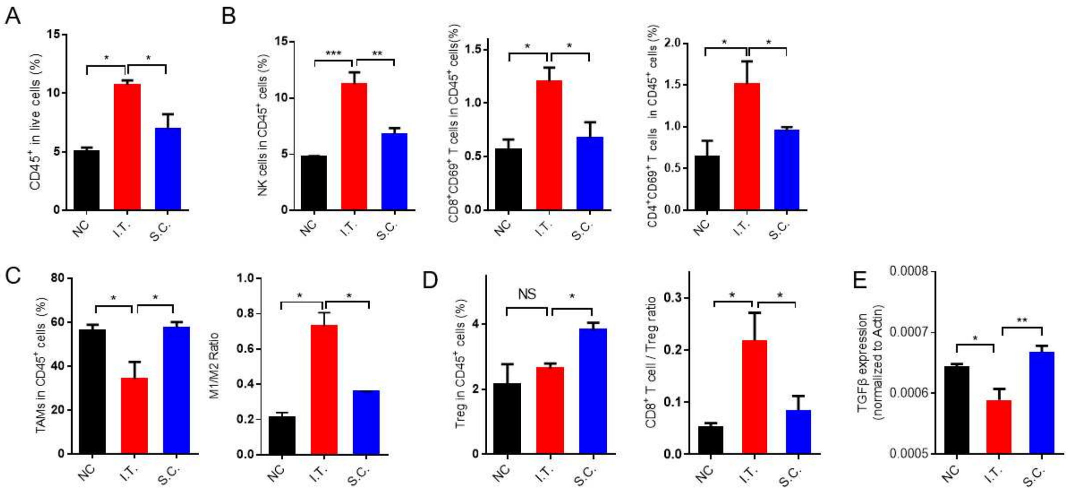

Supplementary Figure 6

Analysis of immune profiles in the tumor microenvironment 7 days after vaccination.

Mice bearing TC-1 tumors were vaccinated I.T. or S.C. (1 μg E7 peptide plus 30 μg PC7A NP per mouse). No treatment (NC) control mice were I.T. injected with PBS buffer. Tumors were made into single cell suspension and analyzed 7 days after vaccination (n= 5). (A) $CD45^+$ cells in tumor after first vaccination. (B) Active lymphocyte subpopulations in tumors, including NK cells ($CD3^-$ $CD49b^+$), $CD69^+CD8^+$ T cells, and $CD69^+CD4^+$ T cells. (C) The percentage of Macrophages ($CD11b^+$ $Ly6G^-$ $Ly6C^-$ $F4/80^+$) and the ratio of M1 subset ($CD11b^+$ $Ly6G^-$ $Ly6C^-$ $F4/80^+$ $MHCII^+$) over M2 subset ($CD11b^+$ $Ly6G^-$ $Ly6C^-$ $F4/80^+$ $MHCII^-$) in tumor were analyzed. (D) The percentage of Tregs ($CD3^+$ $CD4^+$ $FoxP3^+$) and the ratio of $CD8^+$ T cells/Tregs in tumor was analyzed. (E) The expression levels of TGF-β in tumor were measured by qPCR . ***P<0.001, **P<0.01, *P<0.05. NS, not significant, one-way ANOVA t-test.

Jiang X, *et al. J Immunother Cancer* 2022; 10:e003960. doi: 10.1136/jitc-2021-003960

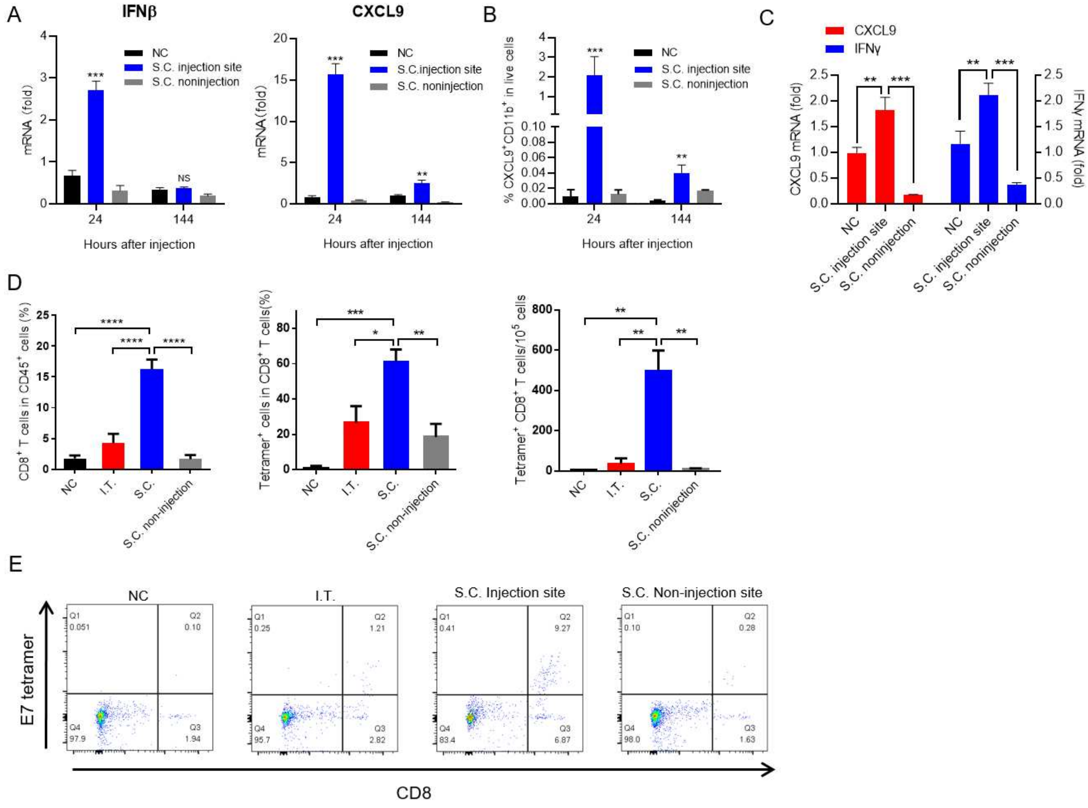

Supplementary Figure 7

S.C. vaccination increases T cell accumulation at the injection site.

Mice were inoculated subcutaneously with TC-1 tumor cells (1.5×10^5 per mouse) into the right flank. Mice received S.C. vaccination (1μg E7 peptide plus 30μg PC7A polymer per mouse) on Days 10, 17 and 24 after tumor inoculation, and S.C. PBS was included as negative control. (A-C) 24h and 144h after first vaccination, subcutaneous tissues of injection site and none-injection site of s.c. vaccinated mice were collected for analysis by Q-PCR and flow cytometry, subcutaneous tissues of PBS injection in the similar place were included as negative control. (A) Quantification of IFNβ and CXCL9 mRNA expression. (B) Percentage of $CXCL9^{+}CD11b^{+}$cells. (C) Quantification of CXCL9 and IFNγ mRNA expression. (D-E) 7 days after the final vaccination, subcutaneous tissues of injection site and none-injection site of s.c.

Jiang X, *et al. J Immunother Cancer* 2022; 10:e003960. doi: 10.1136/jitc-2021-003960

vaccinated mice were analyzed by flow cytometry, subcutaneous tissues of control and i.t. vaccinated mice in the same place were also included. Proportion of $CD8^+$ T cells, $Tetramer^+CD8^+$ T cells and cell counts of E7 specific cells per 10^5 live cells (D). Representative flow dot plots of $H\text{-}2k^b$ /SIINFEKL tetramer and CD8 antibody staining of $CD45^+$ T cells (E). ***P<0.001, **P<0.01, *P<0.05, NS, not significant, one-way ANOVA t-test.

Jiang X, *et al. J Immunother Cancer* 2022; 10:e003960. doi: 10.1136/jitc-2021-003960
