# Supplementary material for: Intratumoral administration of STING-activating nanovaccine enhances T cell immunotherapy
Source: J Immunother Cancer. 2022 May 26;10(5):e003960. doi: 10.1136/jitc-2021-003960 (PMC9150169; doi:10.1136/jitc-2021-003960)
Supplement: Supplementary data [file jitc-2021-003960supp002.pdf]

Intratumoral Administration of STING-Activating Nanovaccine Enhances T Cell Immunotherapy

Xiaoyi Jiang[1,†], Jian Wang[2,7,†], Xichen Zheng[1],Zhida Liu[6], Xinyu Zhang[1], Yuwei Li[1], Jonathan Wilhelm[2], Jun Cao[1], Gang Huang[2], Jinlan Zhang[1], Baran D. Sumer[4], Jayanthi Lea[5], Zhigang Lu[1,3,], Jinming Gao[2,4,*], and Min Luo[1,2,*]*

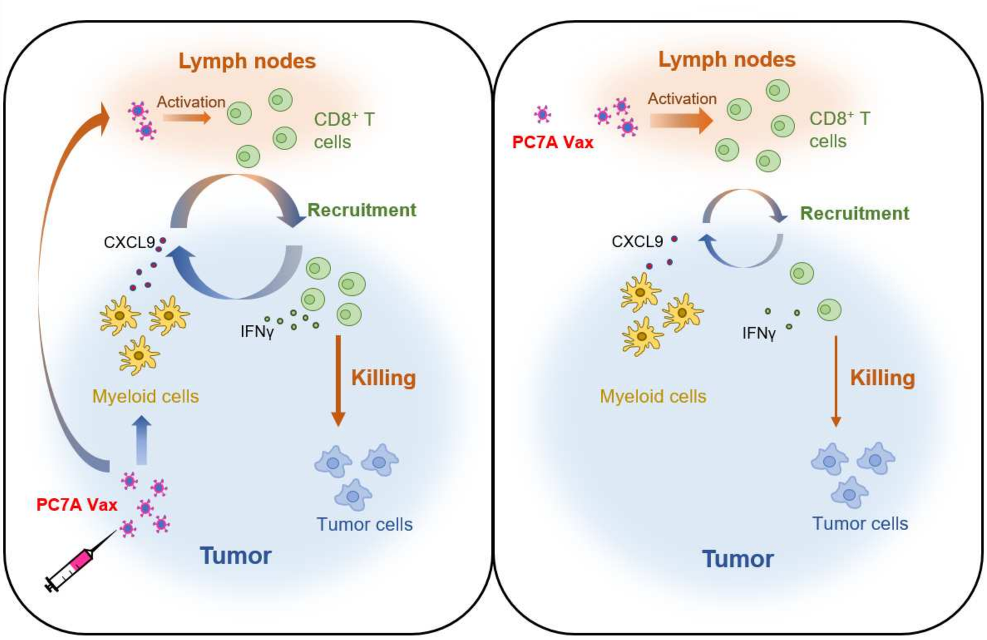

I.T. Vaccination S.C. Vaccination

- **I.T. administration of nanovaccine enhances antitumor efficacy over S.C. injection**
- **I.T. vaccination initiates the myeloid cell/CXCL9-CD8+ T/IFNγ feedback loop for T cell recruitment**
